# Mathematical Modelling Long-Term Effects of Replacing Prevnar7 with Prevnar13 on Invasive Pneumococcal Diseases in England and Wales

**DOI:** 10.1371/journal.pone.0039927

**Published:** 2012-07-13

**Authors:** Yoon Hong Choi, Mark Jit, Stefan Flasche, Nigel Gay, Elizabeth Miller

**Affiliations:** 1 Health Protection Services Colindale, Health Protection Agency, London, United Kingdom; 2 Centre for the Mathematical Modelling of Infectious Diseases, London School of Hygiene and Tropical Medicine, London, United Kingdom; 3 Department of Mathematics and Statistics, University of Strathclyde, Glasgow, United Kingdom; 4 Fu Consulting, Hungerford, Berkshire, United Kingdom; Centers for Disease Control & Prevention, United States of America

## Abstract

**Introduction:**

England and Wales recently replaced the 7-valent pneumococcal conjugate vaccine (PCV7) with its 13-valent equivalent (PCV13), partly based on projections from mathematical models of the long-term impact of such a switch compared to ceasing pneumococcal conjugate vaccination altogether.

**Methods:**

A compartmental deterministic model was used to estimate parameters governing transmission of infection and competition between different groups of pneumococcal serotypes prior to the introduction of PCV13. The best-fitting parameters were used in an individual based model to describe pneumococcal transmission dynamics and effects of various options for the vaccination programme change in England and Wales. A number of scenarios were conducted using (i) different assumptions about the number of invasive pneumococcal disease cases adjusted for the increasing trend in disease incidence prior to PCV7 introduction in England and Wales, and (ii) a range of values representing serotype replacement induced by vaccination of the additional six serotypes in PCV13.

**Results:**

Most of the scenarios considered suggest that ceasing pneumococcal conjugate vaccine use would cause an increase in invasive pneumococcal disease incidence, while replacing PCV7 with PCV13 would cause an overall decrease. However, the size of this reduction largely depends on the level of competition induced by the additional serotypes in PCV13. The model estimates that over 20 years of PCV13 vaccination, around 5000–62000 IPD cases could be prevented compared to stopping pneumococcal conjugate vaccination altogether.

**Conclusion:**

Despite inevitable uncertainty around serotype replacement effects following introduction of PCV13, the model suggests a reduction in overall invasive pneumococcal disease incidence in all cases. Our results provide useful evidence on the benefits of PCV13 to countries replacing or considering replacing PCV7 with PCV13, as well as data that can be used to evaluate the cost-effectiveness of such a switch.

## Introduction

Prior to the introduction of Prevnar 7™, the 7-valent pneumococcal conjugate vaccine (PCV7), in England and Wales, *Streptococcus pneumoniae* was responsible for about 6,000 annual cases of invasive pneumococcal diseases (IPD), with presentations such as meningitis, bacteraemia, sepsis and pneumonia. PCV7 provides protection against 7 serotypes (4, 6B, 9V, 14, 18C, 19F and 23F) among more than 90 pneumococcal serotypes identified so far. It was introduced into the UK routine childhood immunisation programme in September 2006 for infants at 2, 4 and 13 months with a catch up to children under two years of age. Introduction was informed by a dynamic transmission model parameterised using IPD data following PCV7 introduction in the United States. The model predicted elimination of vaccine-type (VT) IPD in the long term with limited serotype replacement, hence leading to sustained reduction in overall IPD in all age groups [1]. However, the post-PCV7 experience in England and Wales has shown more aggressive serotype replacement and less impact on overall IPD than reported in the US, likely due to differences between countries in IPD surveillance systems [2], [3]. An updated analysis using a transmission dynamic model incorporating IPD data from the first three years of the PCV7 programme in England and Wales predicted that in the longer term the reduction in VT IPD due to PCV7 would be largely (though not fully) negated by serotype replacement [4]. In April, 2010, Prevnar 13™ (PCV13) covering extra six serotypes (1, 3, 5, 6A, 7F and 19A) replaced PCV7 in the UK (with no catch-up), sales of PCV7 in the United Kingdom (UK) having been discontinued. Those infants with an incomplete schedule with PCV7 received the remaining doses with PCV13. Hence it is important to understand the likely range of possible short-term effects of PCV13 on disease caused by different serotypes against a backdrop of serotype replacement following PCV7 introduction, as well as its longer term effect on overall IPD incidence.

Here we describe an extension of our previous modelling work in order to address these questions. We use a hybrid modelling approach, combining an enhanced version of the previously developed compartmental deterministic model [1], [4] and its individual based representation to estimate the long-term effect on IPD cases in England and Wales. Two policy options were evaluated: (i) replacing PCV7 by PCV13, and (ii) discontinuing PCV vaccination altogether. Continuing use of PCV7 was not evaluated since the vaccine is no longer available in the UK market. The model results were used to inform the decision on introducing PCV13 in England and Wales in 2011.

## Materials and Methods

### Data

Key model parameters governing transmission and competition between serotypes were determined by fitting to the same epidemiological data, as was done in our previously published model [4]. Data used for this purpose were serotype-specific pneumococcal carriage prevalence acquired from the longitudinal nasopharyngeal swab study conducted in England between 2001 and 2002 [5], trend adjusted serotype specific number of IPD cases between 2005/06 and 2008/09 [2], population mixing information based on a UK contact survey [6], and monthly PCV7 coverage for each birth month [4]. Serotype-specific data were stratified into three groups (as opposed to two in the previous model): the seven serotypes in PCV7 (4, 6B, 9V, 14, 18C, 19F, and 23F; hereafter “VT1” types), the five additional serotypes in PCV13 excluding serotype 1 (3, 5, 6A, 7F, and 19A; “VT2”) and the remaining serotypes in neither vaccine (“NVT”). Serotype 1 was excluded as in previous modelling studies [3] as it has shown large secular changes in incidence in the UK that are unrelated to PCV7 introduction. *S. pneumoniae* in IPD cases was restricted to those identified by culture [2]. The monthly uptake of PCV13 was assumed to be the same as it has been for PCV7.

IPD incidence has been increasing in the years prior to PCV7 introduction, likely reflecting improved ascertainment [4], [7]. For the purposes of estimating parameters governing vaccine impact, we assumed that this trend in IPD has continued for the three years post-PCV7 introduction which was included for model fitting. Three scenarios were used (as previously described [4]), using the midpoint estimate, lower and upper limits of the 95% confidence intervals of the trend analysis and are respectively referred to as “point”, “lower”, and “upper” scenarios in the following.

### Transmission Models

Two types of pneumococcal transmission dynamical models were developed: a deterministic compartmental model to estimate model parameters using the data described in the previous section, and an analogous individual based model to simulate effects of introducing PCV13 or stopping PCV vaccination entirely. Parameters estimated from the deterministic model were used as inputs for the individual based model. The individual based model was used to implement the heterogeneous vaccine/dose/birth cohort information in those individuals who started vaccination with PCV7 but subsequently switched to PCV13 either post dose 1 or post dose 2 during the immunisation course. This was done because the multitude of possible vaccine status and dose combinations is too complex to capture in a compartmental model framework.

### Model Structure – Movement between Compartments

**Figure 1:**
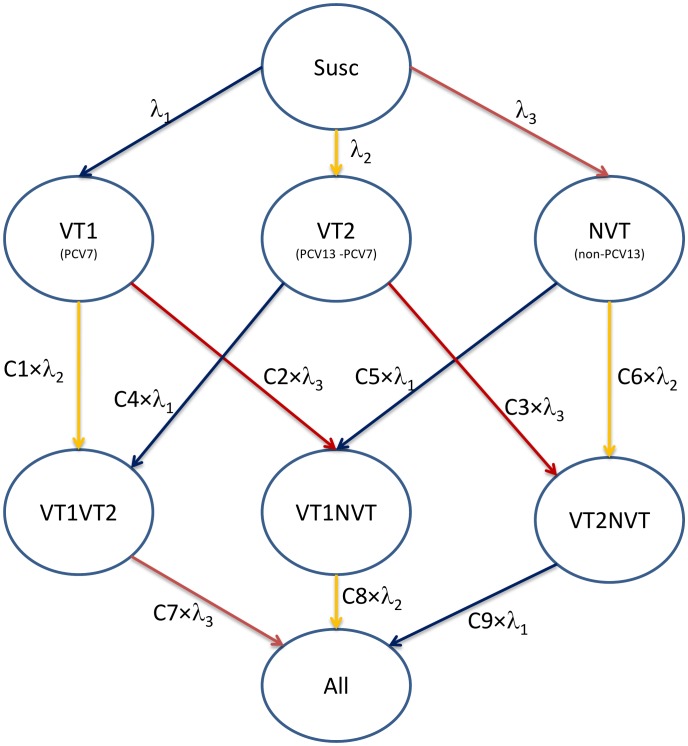
Flow diagram showing the relationship between different states representing carriage of different groups of pneumococcal serotypes in the models used.

Individuals in the population belong to one of eight compartments according to the nature of serotypes they carry in their nasopharynx (deemed an infection): susceptible to any infection (hereafter “Susc”), infected with either VT1, VT2 or NVT serotypes, infected with serotypes from two groups (“VT1VT2”, “VT1NVT”, “VT2NVT”) and infected with serotypes from all three groups (“All”). This is shown graphically in Figure 1. Individuals can clear the infection according to age-dependent clearance rates [4]. Clearance rates were assumed to be similar for all infections (VT1, VT2, and NVT).

Three parameters (λ_1,_ λ_2,_ and λ_3_) in Figure 1 respectively represent the transmission probabilities per close contact for the corresponding three pneumococcal serotype groups (VT1, VT2 and NVT). Nine parameters (C1, …, C9) represent the propensity for competition between three serotype groups: individuals carrying an infection from one serotype group are partially protected against acquiring an infection from another serotype group according to the value of corresponding competition parameter (which ranges between 0 for complete protection and 1 for no competition and no restrictions on coexistence of different serotype groups in individual hosts). For example, individuals carrying VT1 are protected from acquiring VT2 by having the force of infection, λ_3_, reduced by the competition parameter C1 (see Figure 1).

The culture method used for identifying serotypes in carriage samples has poor sensitivity to multiple carriage [8]. Therefore, the competition parameters can only be estimated by fitting the model using post vaccination data showing the magnitude of replacement of VT IPD cases with NVT IPD cases.

1. As in Choi et al. [4], the magnitude of competition induced by carrying the serotypes in PCV7 against other serotype groups (here, C1 and C2) can be estimated by making use of pre- and post- PCV7 vaccination IPD data.
2. Similarly, C3 cannot be estimated since post PCV13 vaccination data was not available at the time of the analysis. However, this parameter, governing competition between NVT and VT2 is highly influential in terms of determining the replacement effects following vaccination with PCV13. For this reason, we explored the whole parameter range by employing three scenarios (C3 = 0, 0.5, and 1).
3. Competition parameters C4 and C5 represent the protection that individuals carrying VT2 or NVT have against acquiring VT1. These parameters do not alter the prevalence of VT2 or NVT after PCV7 introduction since with the elimination of VT1 carriage the respective compartments of multiple carriage disappear. Hence C4 and C5 cannot be estimated using the post-PCV7 data but also the post vaccination epidemiology is insensitive to them. For similar reasons, the competition parameters C6, C7, C8 and C9 also have no significant effect on the post PCV7 and PCV13 epidemiology. We fixed all these competition parameters (C4 to C9) at 0.5.

### Fitting Procedures - Post-PCV7 Data

Model parameters governing transmission and coexistence between different groups of serotypes within individuals were estimated by fitting the deterministic model to IPD data in both the pre-PCV7 and post-PCV7 periods in the following way:

1. The model’s goodness of fit (measured in terms of the Poisson deviance between the prevalence of pneumococcal carriage predicted by the model and reported in the carriage study) is calculated for six age groups among the three serotype groups assuming that the culturing processes only detect the most dominant serogroups amongst the serotypes carried. The order of this dominancy is assumed to follow: VT1>VT2>NVT.
2. The propensity to develop invasive disease given carriage for three serotype groups and 16 age groups is calculated using the trend-adjusted number of IPD cases in 2005/06, assuming carriage prevalence has been stable since 2000/01.
3. The goodness of fit between the number of IPD cases predicted by the model (which is determined by the predicted carriage prevalence and the calculated propensity to develop invasive disease given carriage) and trend-adjusted post-PCV7 data (measured in terms of Poisson deviance) is calculated.
4. A simplex algorithm determining the minimal overall deviance (FMINSEARCH in MATLAB™) was employed to find the optimal parameter sets for the different scenarios.

### Individual Based Model

The dynamics of pneumococcal transmission and the likely impact of changing the valency of the PCV vaccination programme were modelled in an individual based framework that exactly reproduced the deterministic model. The simulations were run with four time steps for each month, with 100 age cohorts from 0 to 99 year olds. As a consequence, the total population size is 48 million, comprising of 10,000 individuals in each of 4,800 birth cohorts. As in the deterministic model each age cohort is weighted based on the population structure of England and Wales in 2006 to estimate the force of infection for three serotype groups at each time step [4]. Hence the overall weighted population size gives a realistic representation of the number of IPD cases in England and Wales. At each time step, 10,000 individuals in the last weekly age cohort are removed and replaced with 10,000 susceptible individuals in the first weekly age cohort, representing a simple birth and death process [4].

Acquisition and loss of infection, and loss of vaccine protection occur as stochastic processes, with their probabilities determined by the force of infection, duration of infection, and duration of vaccine protection, respectively. Each scenario is repeated ten times in order to estimate the stochastic uncertainty arising from these processes.Only ten repetitions were necessary as the large population size (48 million) limited stochastic variation between repetitions and only their mean is reported here.

### Vaccination Scenarios in the Individual Based Model

**Figure 2:**
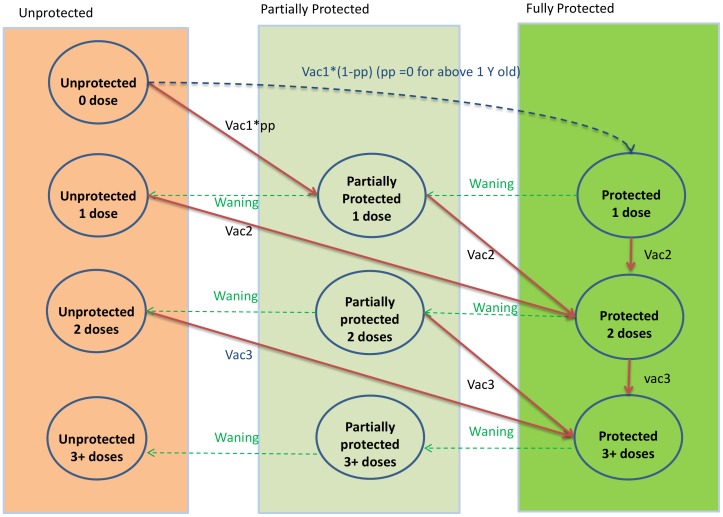
Flow diagram showing how vaccination status of individuals is tracked in the model, based on the number of doses received and waning of vaccine protection. Individuals who receive one dose while above the age of one year are fully protected. Those who receive their first dose under the age of one year are only partially protected. Those who receive 2 or more doses receive full protection. Those who lose vaccine protection move to the next less well protected group.

The effect of each dose of the PCV vaccination programme in the individual based model is shown in Figure 2:

1. Individuals who receive one dose when above the age of one year are fully protected. Those who receive one dose under the age of one year are only partially protected.
2. Individuals who receive two or more doses are fully protected.
3. Individuals who lose vaccine protection by waning are only partially protected.
4. If individuals who are partially protected receive another dose, they then become fully protected.
5. PCV7 vaccination begins in September 2006 and is either changed to PCV13 or stopped in April 2010.
6. When PCV7 is replaced with PCV13, individuals who were scheduled to receive the remaining PCV7 doses receive PCV13 instead and get protection against extra serotypes included in PCV13. For example, if an infant received the first dose with PCV7 and the second dose with PCV13, the infant gets full protection against PCV7 serotypes but only partial protection against extra serotypes in PCV13.

### Vaccine Efficacy Against Disease Development and Infection

The duration of vaccine protection against acquisition of carriage and disease development is assumed to be 5 years [4]. The degree of vaccine protection for vaccinated individuals against acquiring VT1 and VT2 carriage is assumed to be 52% for fully protected individuals and 26% for partially protected individuals [4]. A similar figure was estimated by Rinta-Kokko et al. [9]. Vaccine efficacy against developing IPD is assumed to be 100% for both partially and fully protected individuals, based on fitting to data and as assumed in previous studies [1], [4]. PCV7 and PCV13 were assumed to be similar in those aspects.

## Results

### Fitting

Table 1 presents the best fitting pairs of the competition parameters, C1 and C2, representing the protective effect of carrying VT1 infections against acquiring infections with serotypes belonging to VT2 and NVT respectively. In all scenarios, protection against both serogroups exceeds 58%.

**Table 1:** Competition parameters C1 and C2 for individuals carrying VT1 serotype infections who get protection against acquiring carriage due to VT2 and NVT serotypes.

C3	0			0.5			1		
Trend	Point	Lower	Upper	Point	Lower	Upper	Point	Lower	Upper
C1	0.25	0.20	0.37	0.29	0.21	0.40	0.27	0.20	0.39
C2	0.02	0.00	0.20	0.26	0.17	0.41	0.17	0.08	0.33

C3 (the competition parameter representing the protective effect of carrying VT2 against acquiring NVT serotypes) takes values of 0, 0.5 and 1 in different scenarios. The IPD data were adjusted using three scenarios about the year-on-year increase in the number of IPD cases based on pre-PCV7 trends.

**Figure 3:**
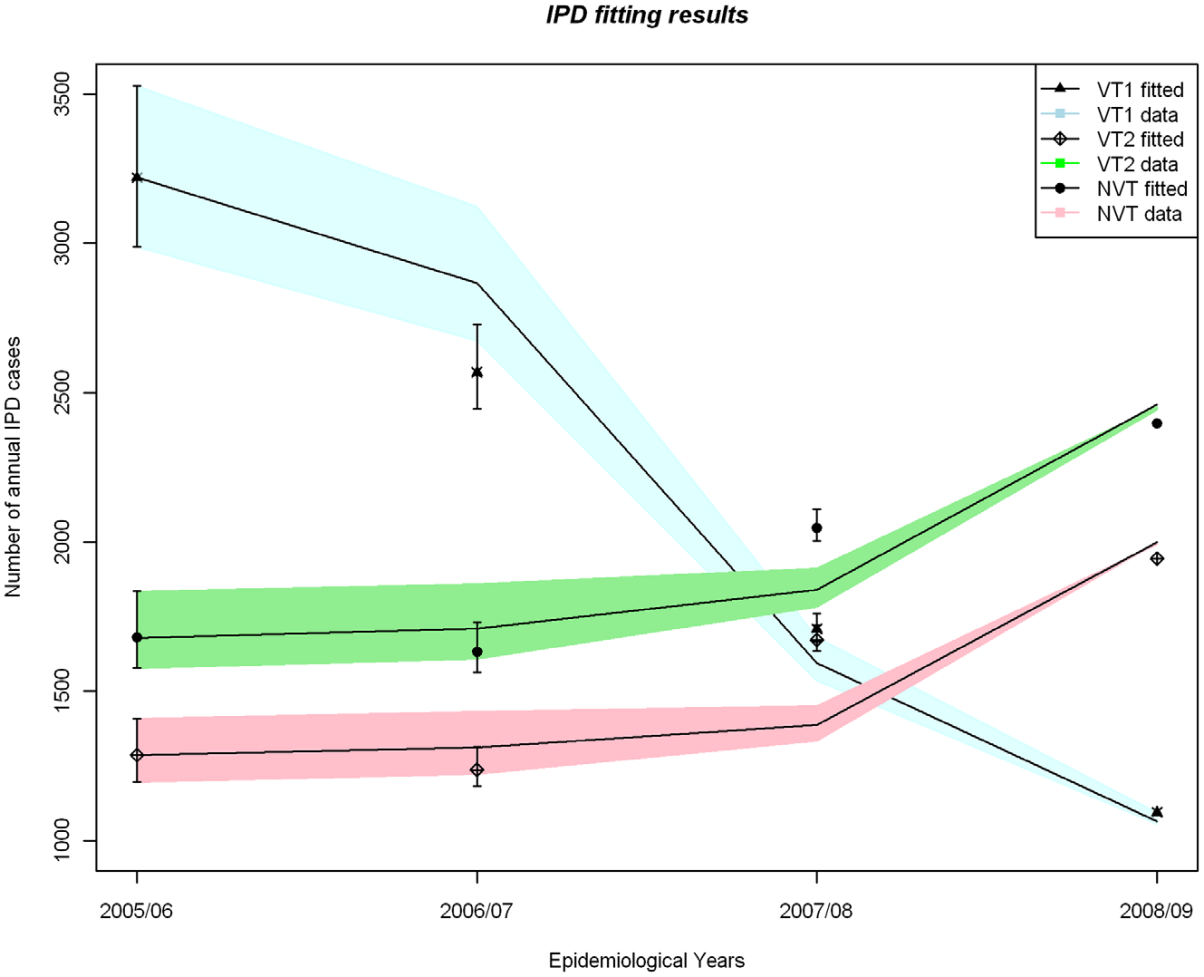
Comparison between the annual number of IPD cases reported pre and three years post- PCV7 introduction, with fitting results based on the model when C3 (representing the protective effect of carrying VT2 serotypes against infection with NVT) is fixed at 0.5. The number of IPD cases has been adjusted using the upper, lower and medium scenarios for the secular trend in IPD prior to PCV7 introduction.

For all three scenarios of data adjustment for trend, the model fitted the number of IPD cases as observed in England and Wales well (Figure 3). In all these scenarios vaccination with PCV7 from September 2006 resulted in a rapid reduction in PCV7 serotypes and a concurrent increase in both VT2 and NVT serotypes.

### Long-term Effects on IPD Cases

**Figure 4:**
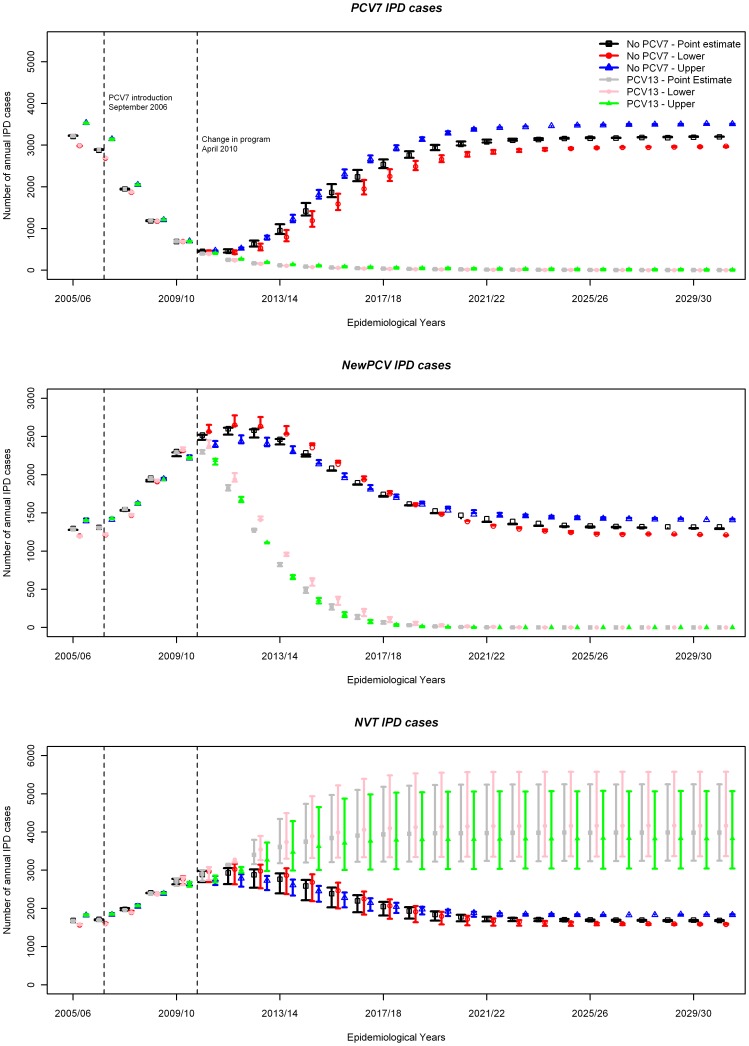
Model projections of the long-term effects of either stopping pneumococcal conjugate vaccination or replacing PCV7 with PCV13 on April 2010 on the number of IPD cases caused by VT1, VT2, and NVT serotype groups in England and Wales over 35 years since the introduction of PCV7. C3 (representing the protective effect of carrying VT2 serotypes against infection with NVT) takes the values 0, 0.5, and 1 in different scenarios. Filled circles show results with C3 = 0.5 and the error bars represent results with C3 = 0 or 1. Scenarios are presented in terms of IPD data being adjusted by high, low and medium estimates for the secular trend in IPD prior to PCV7 introduction.

**Figure 5:**
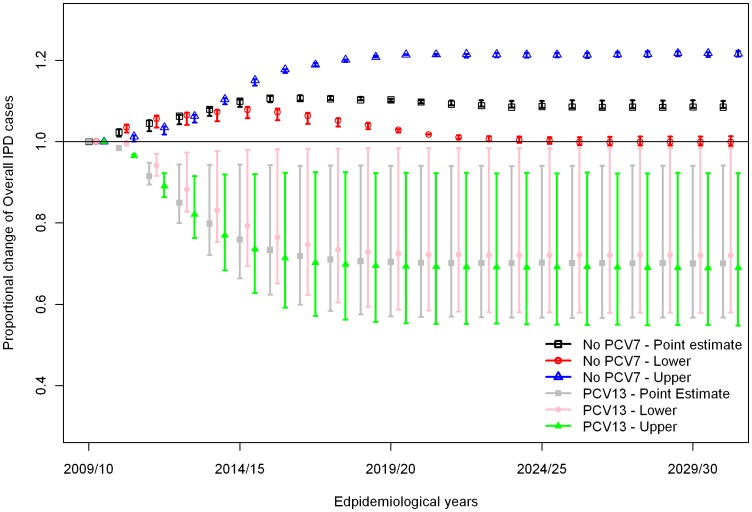
Proportional changes in the number of overall IPD cases following either stopping pneumococcal conjugate vaccination or replacing PCV7 with PCV13 on April 2010. PCV7 is replaced with PCV13 six years after PCV7 introduction. C3 (representing the protective effect of carrying VT2 serotypes against infection with NVT) takes the values 0, 0.5, and 1 in different scenarios. Filled circles show results with C3 = 0.5 and the error bars represent results with C3 = 0 or 1. IPD data were adjusted based on the high, low and medium estimates for the secular trend in IPD prior to PCV7 introduction (corresponding to the upper and lower limits of the error bars as well as the points).

**Figure 6:**
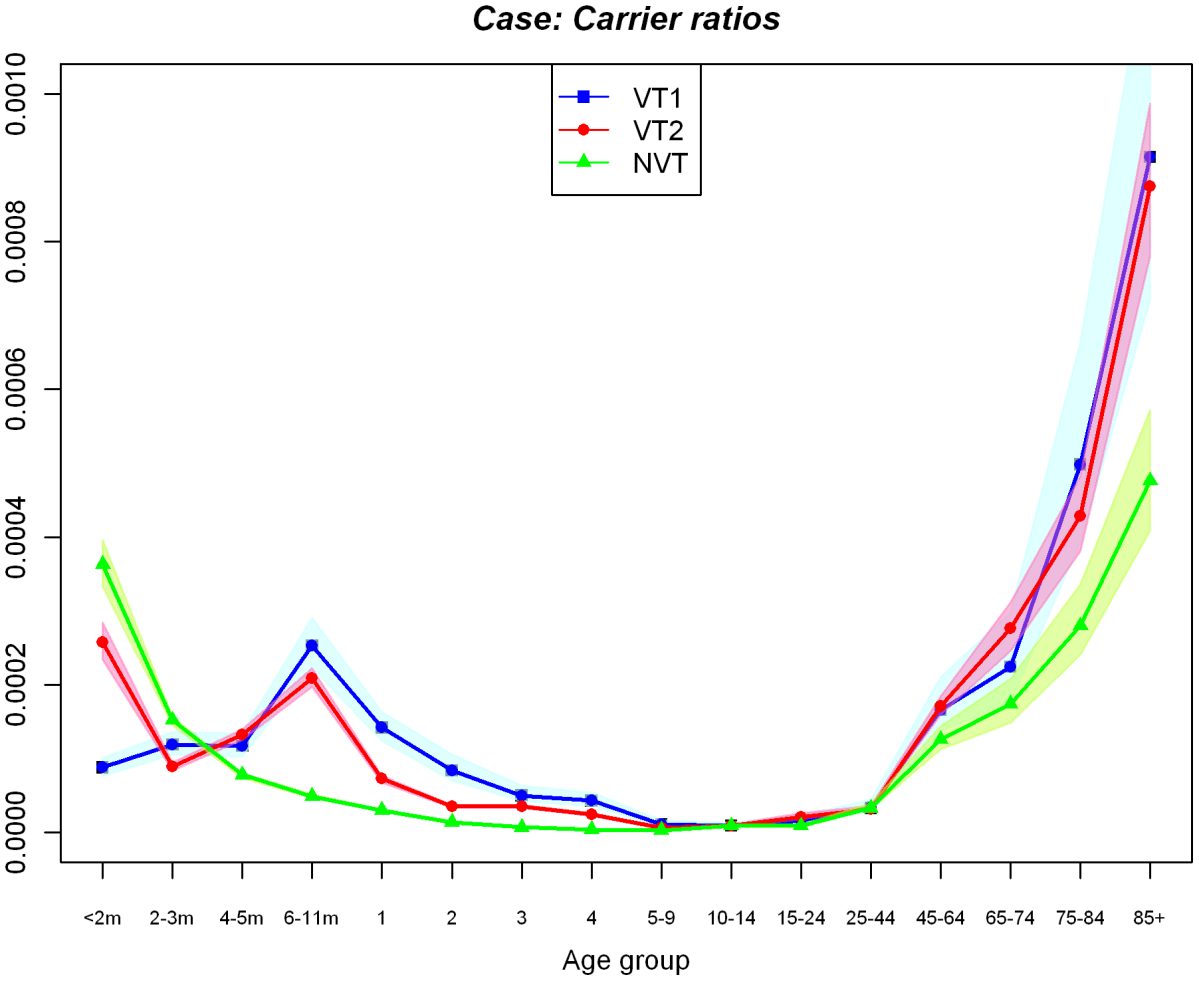
Propensity for individuals carrying pneumococcal serotypes in three serogroups (VT1, VT2 and NVT) among 16 age groups to develop disease. The ratios are obtained from the scenario using 0.5 as the competition parameter, C3, and the IPD cases adjusted using the point estimate of the adjustment for the increasing trend in such cases (shaded areas show the ratios estimated using the lower and upper boundaries of its 95% CIs).

Stopping PCV vaccination altogether in April 2010 is predicted to cause a rebound in the number of IPD cases to the pre-PCV7 equilibrium due to an increase in VT1 IPD as well as decreases in both VT2 and NVT IPD (Figure 4). On the other hand, a reduction in the number of IPD cases due to VT2 serotypes is predicted if PCV13 is introduced from April 2010, eventually leading to elimination of both VT1 and VT2 serotypes, even though there is no immediate reduction in VT2 IPD cases among unvaccinated individuals in the first few years due to ongoing replacement effects from PCV7 in the population. With PCV13 introduction, the number of NVT IPD cases increases regardless of the value of the competition parameter, C3, because of the ongoing serotype replacement effect caused by PCV7 introduction. However, stronger competition (lower C3) would cause much larger increases in the number of NVT IPD cases (Figure 4) which leads to a smaller reduction in overall IPD (Figure 5). The reduction in the overall number of IPD cases with the introduction of PCV13 in all scenarios considered is due to the lower case: carrier ratios of non-PCV13 serotypes compared to those in VT1 and VT2 serogroups in elderly age groups where the most IPD cases occur (Figure 6).

The baseline epidemiological year for calculating the impact of the change in vaccination policy away from PCV7 is assumed to be 2009/10, since PCV13 was only implemented three months before the end of 2009/10. Table 2 presents the difference in model projections of the cumulative and annual numbers of IPD cases after PCV13 introduction over the 20 years from baseline to the epidemiological year 2029/30, compared to no PCV vaccination. Introduction of PCV13 is estimated to reduce the number of IPD cases in the subsequent 20 years by 5,000 to 62,000 compared to the scenario of discontinuing PCV7. The difference in the annual number of IPD cases in the epidemiological year 2029/30 ranges from 180 (6% of the number predicted without PCV vaccination) to 3700 (42%) compared to the epidemiological year 2009/10 depending on the scenarios governing the IPD trend and the value for the competition parameter C3.

**Table 2:** Model estimates of the amount of prevented cases by introducing PCV13 compared to discontinuing PCV7.

Competition	C3 = 0			C3 = 0.5			C3 = 1		
Trend	Point	Lower	Upper	Point	Lower	Upper	Point	Lower	Upper
Cumulative differencein cases over 20 years	16,664	5,346	28,114	39,324	30,092	49,394	51,231	43,915	62,851
Annual difference	928	182	1,675	2,213	1,603	2,927	2,934	2,397	3,700

The analysis is shown for both the cumulative difference in cases between 2009/10 to 2029/30 and the difference 20 years after the change in policy (2029/30).

## Discussion

PCV13 replaced PCV7 in the UK’s vaccination schedule in April 2010 following the manufacturer’s decision to cease marketing PCV7 in the UK and only to offer PCV13. At the time of the analysis neither information about multiple carriage episodes nor data on post PCV13 IPD replacement was available. Such information is needed to estimate the competition parameter determining the protection individuals carrying VT2 receive against acquiring NVT infections and the consequent level of NVT replacement expected following the introduction of PCV13. However, we were able to capture this uncertainty and show that even in the case of strong competition between those serotype groups, the corresponding level of serotype replacement is unlikely to offset the benefits in reduction in IPD, which is mainly due to the lower invasiveness of the NVT serotype group among over 65 year olds where the majority of IPD cases occur (Figure 6).

The indirect impact of pneumococcal conjugate vaccines is governed by two opposing effects. The beneficial effect is the reduction in the number of vaccine related IPD cases among unvaccinated individuals in the population due to the reduction in pneumococcal carriage and transmission in vaccinated individuals. The detrimental effect is the replacement of vaccine types with non vaccine types: removing the targeted serotypes leaves open an ecological niche for other serotypes to colonise. When there is little propensity for different serotypes to coexist in the host then it is assumed that there is competition between them to colonise the nasopharynx, so carrying one serotype has a protective effect against acquiring another serotype. The stronger the effect of this competition (with a consequent lower propensity to coexist), the more likely it is that individuals will acquire NVT serotypes following vaccination because competition induced by vaccine serotypes is reduced. The existence of such competition is widely accepted [3] and is supported by the increase in NVT IPD cases in England and Wales since PCV7 introduction [2]. Using mathematical models we found that the parameters governing competition between serotypes in PCV13 and other serotypes is crucial for estimating replacement following its introduction and thereby the overall effects on pneumococcal invasive disease.

The mechanisms driving competition and co-existence between the various pneumococcal serotypes are poorly understood but play a crucial role in determining the effects of vaccination on the transmission dynamics of pneumococcal carriage and disease. The modelling approach we implemented accounts for both these aspects with a mechanistic rather then a causal representation of competition between serotypes. The model promotes co-existence of serotypes trough implicitly assuming serotype group specific immunity for the duration of carriage and thus providing an ecological niche for other serotype groups. Hence, the model is not structurally neutral [10]. Despite this potential shortcoming, by fitting just a few parameters (competition and propensity to cause disease when colonised) the model was able to closely reproduce the observed disease due to VT1, VT2 and NVT serotypes before introduction of PCV13 as well as the observed herd immunity and non-vaccine-type replacement in the PCV7 era between 2005/6 and 2008/09. Hence, in the absence of further knowledge (in particular of observed post PCV13 replacement) this model suggests a likely range of impact following the change in pneumococcal vaccination policy in April 2010 in the UK.

In this paper, we present a novel model to estimate *a priori* the effect on IPD of replacing PCV7 vaccination with pneumococcal vaccines of higher valency (PCV13) that captures the transmission dynamics of herd immunity and serotype replacement. In order to capture the effect of giving different vaccines (PCV7 and PCV13) to the same individuals, we used an individual based modelling framework that allowed detailed characteristics of each individual within a model to be tracked. However, the computational requirements of this choice of model structure make it challenging to estimate model parameters in a complex dynamical system. Hence we used a hybrid approach, with a deterministic compartmental transmission dynamic model used to estimate relevant model parameters using pre-PCV7 carriage data, trend-adjusted IPD data between pre- and post-PCV7 periods, age-dependent mixing and PCV7 coverage. Once these parameters had been estimated, we used an individual based model in order to measure the effects of two alternative vaccination programmes on the number of annual IPD cases.

One encouraging result is that all the scenarios considered in this study suggest reductions in the overall number of IPD cases following PCV13 introduction, though of varying magnitude. This predicted reduction in the overall number of IPD cases following PCV13 introduction is due to the lower propensity to develop invasive disease given carriage of non-PCV13 serotypes in elderly age groups where most of the IPD cases occur. Similar results were found in a UK study predominantly in children [11]. Furthermore, our estimates are likely to be conservative since we do not account for the highly invasive serotype 1. The scenario we examine that predicts the largest reduction in the number of IPD cases following PCV13 introduction (compared to stopping PCV vaccination altogether) occurs in the absence of competition between the additional serotypes in PCV13 and non-PCV13 serotypes which would have caused no serotype replacement (see Table 2), reflecting an assumption that there is perfect coexistence between VT2 and NVT serotypes in individual hosts. This appears unlikely since the protective effect of carriage on heterologous acquisition of carriage was found to be high for various settings [4], [12]. If this also holds true for the additional serotypes in PCV13, then the effect of PCV13 introduction on pneumococcal carriage will be largely negated in older age groups by the replacement of VT2 serotypes with NVT serotypes, as was the case following PCV7 introduction in England and Wales [4]. However, PCV13 may still be beneficial in terms of IPD reduction since the NVT serotypes have a lower propensity to develop invasive disease compared to VT2 serotypes.

Many high income countries with existing immunisation programmes using PCV7 are replacing or considering replacing PCV7 with PCV13 due to its increased serotype coverage. However, this change will not eliminate the potential for serotype replacement since most of the pneumococcal serotypes causing IPD are not present in PCV13 either. Hence the medium and long term effects of using PCV13 in the context of pre-existing use of PCV7 are still largely unknown. Several models have been developed to estimate this effect [13]–[15]. However existing published models are static; hence they make assumptions about serotype replacement following PCV13 introduction that are not informed by the dynamics of intertype competition. In contrast we here present a dynamic model capturing the possible range of values for parameters governing serotype replacement.

**Figure 7:**
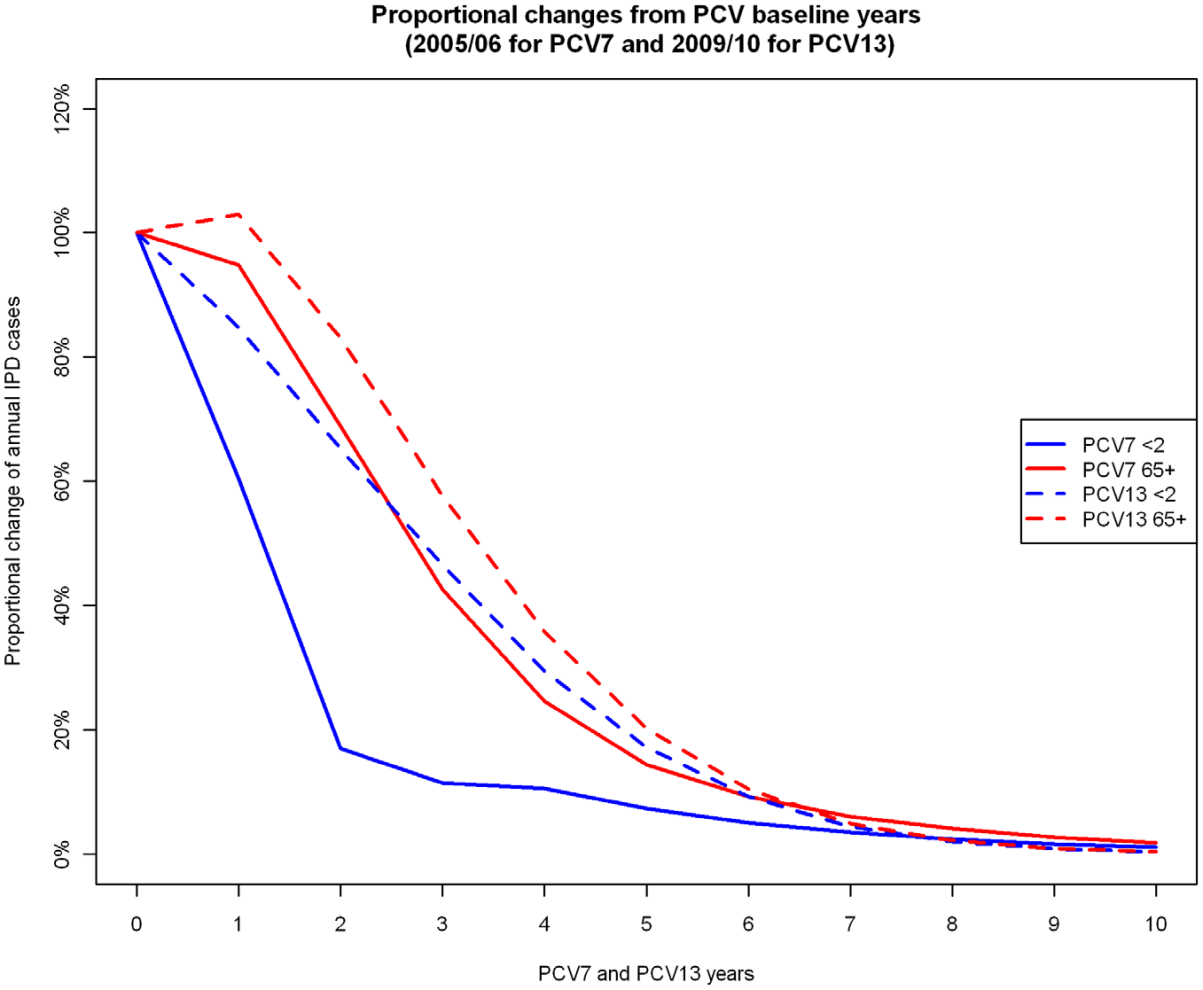
Annual proportional changes in the number of overall IPD cases among age groups aged <2 and ≥65 from two PCV baseline years (epidemiological year 2005/06 for the PCV7 baseline and epidemiological year 2009/10 for the PCV13 baseline).

This study uses dynamic (deterministic and individual based) models to estimate the direct and indirect effects of replacing an existing infant PCV7 vaccination programme with a higher valency vaccine such as PCV13. The transmission model allows more realistic estimation of dynamic effects (indirect protection and serotype replacement) of pneumococcal conjugate vaccines than static models, even when the latter are adjusted using a “dynamic approximation” technique [16]. We find that in the short term, replacement of PCV7 with PCV13 is not likely to bring an immediate rapid reduction in overall IPD cases, because replacement of VT1 serotypes with VT2 and NVT serotypes is still progressing among unvaccinated individuals (Figure 7). However in the long term, a substantial reduction in IPD cases is likely to occur.

### Conclusions

In this study we estimated parameters governing the effect of introducing PCV13 to replace PCV7 or of discontinuing pneumococcal conjugate vaccination through parsimonious dynamic models. Prior to robust data on replacement induced by vaccination with PCV13 and in the absence of information about multiple carriage episodes, a key aspect of the impact of the introduction of PCV13, serotype replacement from the reduced competitive effect of the six additional vaccine serotypes, could not be estimated. However, we show that the possible range of the replacement effect –although substantial- is unlikely to span scenarios where introduction of PCV13 becomes less beneficial than the alternative option of discontinuing conjugate vaccination. Data on multiple carriage would help to reduce the uncertainty of such a priori estimations but would be difficult to obtain because of the large sample size needed to adequately estimate age-dependent carriage prevalence for the compartments of multiple carriage defined in the model. Hence our results provide useful evidence on the possible benefits of PCV13 to countries replacing or considering replacing PCV7 with PCV13, as well as data that can be used to evaluate the cost-effectiveness of such a switch.
